# A Novel Phenolic Resin Aerogel Modified by SiO_2_-ZrO_2_ for Efficient Thermal Protection and Insulation

**DOI:** 10.3390/gels11121018

**Published:** 2025-12-18

**Authors:** Yifan Zhan, Chunhui Zhang, Liangjun Li, Mengle Huang, Sian Chen, Yonggang Jiang, Junzong Feng, Yijie Hu, Jian Feng

**Affiliations:** 1 State Key Laboratory of Pulp and Paper Engineering, South China University of Technology, Guangzhou 510640, China; 17839767768@163.com (Y.Z.); 15067708540@163.com (M.H.); 2 Science and Technology on Advanced Ceramic Fibers and Composites Laboratory, College of Aerospace Science and Engineering, National University of Defense Technology, Changsha 410073, China; chensian07@nudt.edu.cn (S.C.); jygemail@nudt.edu.cn (Y.J.); junzongfeng@nudt.edu.cn (J.F.); hyjscut@163.com (Y.H.); fengj@nudt.edu.cn (J.F.)

**Keywords:** phenolic resin, inorganic–organic composite aerogels, high-temperature resistance, thermal insulation

## Abstract

Phenolic aerogel holds great promise for applications in thermal protection against ablation, and constructing inorganic–organic hybrid networks is an effective strategy to enhance its oxidation and ablation resistance. This study introduces a stepwise hybridization strategy for the preparation of SiO_2_–ZrO_2_–phenolic resin aerogels (SZPA). First, nano-silica sol and nanometer-scale zirconia were physically blended to form a uniformly dispersed mixture. Subsequently, the modified silica was incorporated into a phenolic resin solution to construct a three-dimensional hybrid silica–phenolic network framework. Nano-sized zirconia was then uniformly dispersed within the matrix as a physical reinforcing phase through high-shear dispersion. Finally, the SZPA with a hierarchical nanoporous structure was obtained via ambient-pressure drying. Owing to its unique hybrid network structure, the aerogel exhibits markedly improved properties: the thermal conductivity is as low as 0.0419–0.0431 W/(m·K) (a reduction of approximately 24%), and the specific surface area is as high as 190–232 m^2^/g (an increase of approximately 83%). Meanwhile, the inorganic network considerably enhances the residual mass at elevated temperatures, as well as the oxidation resistance and thermal stability of the matrix. Among the tested materials, the SZPA-4 exhibited outstanding thermal insulation capability at high temperatures; its back surface temperature reached only 74.4 °C after 600 s of exposure to a 1200 °C butane flame. This study provides a feasible route for the preparation of high-performance phenolic-based composite aerogels for aerospace thermal protection systems, thereby expanding their potential applications in extreme thermal environments.

## 1. Introduction

As next-generation spacecraft continually pursue advancements in flight velocity and extended mission durations, they concurrently experience more severe aerodynamic heating on their surfaces. To meet the demands of these extreme operational environments, there is an urgent need to develop high-performance thermal protection materials. Compared to traditional thermal protection materials, aerogels—including silica aerogel [1], alumina aerogel [2,3], carbon aerogel [4], phenolic aerogel [5], polyimide aerogel [6], and polybenzoxazine aerogel [7]—are considered ideal candidate materials owing to their low density, thermal conductivity, and high porosity.

In contrast to inorganic aerogels, organic aerogels have garnered increasing interest owing to their excellent mechanical properties in recent years. Among them, phenolic resin aerogels are particularly notable for their high thermal insulation performance, high mass remaining rate, and low cost, rendering them a promising candidate for ablative thermal protection systems. Since its development by National Aeronautics and Space Administration (NASA), the Phenolic Impregnated Carbon Ablator (PICA)—a lightweight phenolic resin-based ablator—has been a successful thermal protection solution for numerous planetary exploration missions over the past thirty years [8]. However, phenolic resins undergo thermal cracking at elevated temperatures and oxidation in oxygen-rich environments, which degrade their micro-nanoporous structures and lead to a significant deterioration in thermal insulation performance. With the continuous development of functional aerogel materials and the expansion of their application scope, single-component organic or inorganic aerogels have become increasingly inadequate for meeting the specialized functional requirements under extreme aerospace conditions. Therefore, hybrid modification of aerogels has emerged as an important direction in aerogel material development.

Inorganic nanomaterials such as SiO_2_, B_2_O_3_, and ZrO_2_ are commonly employed to enhance the structural stability and ablation resistance of phenolic aerogels. During the ablation process, low-melting-point oxides melt and form a protective viscous liquid phase. This liquid phase infiltrates the pores of the porous char layer, coating both the char layer and the reinforcing fiber surfaces. This coating effectively blocks oxygen diffusion into the composite material, thereby mitigating oxidation and ablation effects. Concurrently, the viscous liquid phase shields the surface char layer and diminishes the impact of aerodynamic shear forces, consequently improving the overall ablation resistance of the composite [9]. High-melting-point oxides (e.g., ZrO_2_) infused into carbon/phenolic composites can form enriched surface layers on the char layer during ablation. Leveraging the low thermal conductivity of ZrO_2_ reduces heat transfer into the material’s interior while simultaneously enhancing the strength of the char layer, thereby improving the composite’s resistance to aerodynamic erosion [10].

Modification methods for phenolic resin aerogels are primarily categorized into two types: physical blending and chemical modification. Physical blending is low-cost and has a short processing time, but it faces challenges in achieving uniform dispersion. Typically, inorganic ceramic fillers containing Si, Zr, and Ti elements are incorporated to enhance ablation resistance [11,12,13]. During ablation, these fillers form a protective ceramic layer on the material surface, which mitigates heat flux and improves ablation resistance. In contrast, chemically modified aerogels demonstrate excellent dispersibility and high structural uniformity; however, their synthesis involves complex processes, higher costs, and potential side reactions. This approach usually incorporates ablation-resistant elements such as B, Si, Zr, and P into the phenolic resin aerogel matrix via chemical bonding [5,14,15,16,17,18,19,20,21]. The introduction of inorganic components effectively enhances the structural integrity of phenolic aerogels while simultaneously improving their ablation and oxidation resistance. Xiao et al. [22] used molecular dynamics simulations to demonstrate the mechanism by which silicon-hybridized phenolic resin aerogels enhance oxidation resistance. The chemical bonding between the phenolic resin and silicon-containing functional groups immobilizes molecules within the aerogel network and suppresses thermal degradation. The formation of silicon-rich clusters is the primary reason for the improved oxidation resistance; however, silicon hybridization alone cannot fulfill more demanding high-temperature requirements. Fu et al. [23] utilized a sol–gel approach to synthesize silica-zirconia hybrid phenolic aerogel. The resulting aerogels demonstrated stable porous architectures and exceptional hydrophobicity. Furthermore, the silicon–zirconium hybridization significantly enhanced the high-temperature char yield and oxidation resistance of the phenolic resins. The aerogel composites also exhibited outstanding thermal protection and insulation properties. The ablation resistance, facilitated by a synergistic Si-Zr effect that generates a protective multiphase ceramic layer (C, SiO_2_, SiC, ZrO_2_), positions this hybrid system as a competitive matrix for lightweight ablative composites. However, in the process of multi-element hybridization, the amount of addition affects the performance of the material.

In this study, SiO_2_–ZrO_2_–phenolic resin aerogels (SZPA) were synthesized via a stepwise hybridization strategy. A three-dimensional hybrid network of silica and phenolic resin was constructed, within which nano-zirconia was uniformly dispersed as a physical reinforcing phase using high-shear mixing. The impact of zirconia contents on the structure and properties of aerogels was studied. This approach effectively addresses the issue of rapid gelation with the phenolic resin matrix, eliminates the cumbersome steps of precursor preparation/modulation and stringent reaction condition control, and thus achieves simplification and cost reduction in the preparation process. The resulting SZPA exhibited remarkable enhancements in aerogel performance: thermal conductivity was reduced to 0.0419–0.0431 W/(m·K) (a reduction of ~24%), while specific surface area increased to 190–232 m^2^/g (an increase of ~83%). Simultaneously, the inorganic network significantly enhanced the matrix’s high-temperature mass remaining rate, oxidation resistance, and thermal stability. Among the samples, SZPA-4 demonstrated outstanding high-temperature insulation performance, maintaining a back-surface temperature of only 74.4 °C after 600 s of exposure to a 1200 °C butane flame.

## 2. Results and Discussion

### 2.1. Synthesis Process

**Figure 1:**
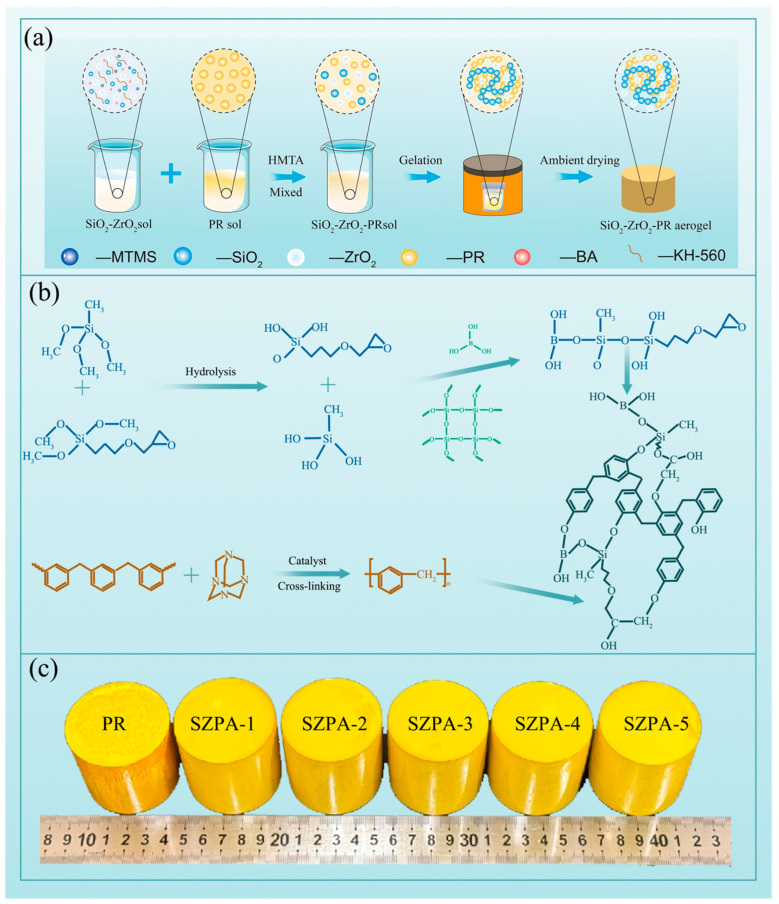
The preparation of SZPA: (**a**) the preparation flow chart of SZPA, (**b**) the synthesis mechanism of SZPA, (**c**) physical photos of SZPA prepared with different zirconia content.

It can be seen from Figure 1a that the preparation process of SiO_2_–ZrO_2_–phenolic resin aerogels (SZPA) includes sol–gel and atmospheric pressure drying. During the sol–gel stage, the silica sol was modified with Methyl trimethoxysilane (MTMS) and γ-(2,3-epoxypropoxy)propytrimethosysilane (KH-560) to modulate its reactivity toward Phenolic resin (PR) sol and regulate the gelation rate. The modification mechanism was schematically illustrated in Figure 1b [24,25]. In Step 1, the methoxy groups (Si–O–CH_3_) in MTMS and KH-560 underwent hydrolysis, yielding silanol groups (Si–OH). Step 2: These silanol groups subsequently condensed with surface hydroxyl groups on silica nanoparticles. Meanwhile, boric acid was introduced to provide boronic hydroxyl groups (B–OH), which condensed with the –OH groups of both silica and phenolic resin, thereby reinforcing the hybrid gel framework [26]. Upon heating, Hexamethylenetetramine (HMTA) decomposes to release formaldehyde and ammonia, acting as a crosslinker and a basic catalyst, respectively. Under the catalysis and crosslinking of HMTA, PR forms organic aerogels with a crosslinked network structure [27]. Concurrently, KH-560 and boric acid facilitated cross-linking between the PR and silica sol particles, while zirconia primarily functioned as a reinforcing filler within the gel network. Zirconia primarily acts as a filler within the gel network. Subsequent ambient-pressure drying yielded SZPA with morphologically intact structures and no observable cracks (Figure 1c). These results confirm the effectiveness of this method for preparing SZPA.

### 2.2. Morphology and Structure

**Figure 2:**
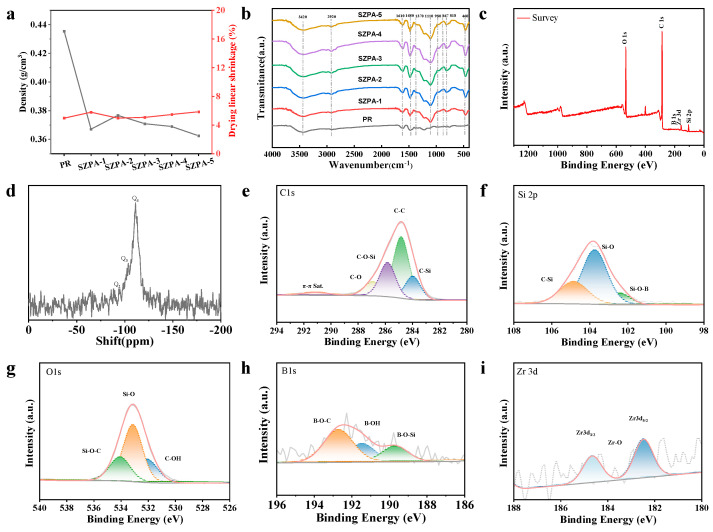
(**a**) PR and SZPA density and linear shrinkage. Chemical structure characterization of PR and SZPA: (**b**) FTIR of PR and SZPA, (**c**) XPS full spectrum scanning of SZPA-4, (**d**) solid-state ^29^Si nuclear magnetic resonance spectrum of SZPA-4, XPS fine spectra of C1s, Si2p, O1s, B1s, and Zr3d orbitals of (**e**–**i**) SZPA-4.

The densities and linear drying shrinkage rates of the PR and SZPA were shown in Figure 2a. The measured densities of PR, SZPA-1, SZPA-2, SZPA-3, SZPA-4, and SZPA-5 were 0.436, 0.367, 0.377, 0.371, 0.369, and 0.363 g/cm^3^, respectively. With the incorporation of silica and zirconia, the drying linear shrinkage of the SZPA remained stable at approximately 5%. The linear shrinkage of aerogels during drying focuses on one-dimensional variation, and the radial dimensional change is selected to characterize the linear shrinkage variation in aerogels. The tests were performed in triplicate. In addition, all samples remained intact without fragmentation after atmospheric pressure drying. This structural integrity is attributed to the stable polymeric network, which effectively withstands the capillary stresses generated during the drying process. These results demonstrated that the proposed method facilitated the preparation of inorganic–organic hybrid aerogels.

The chemical structure of the SZPA was characterized by Fourier transform infrared (FTIR), X-ray Photoelectron Spectroscopy (XPS), and Nuclear Magnetic Resonance (NMR), with the results presented in Figure 2b–i. Figure 2b shows the FTIR spectra of the pristine PR and the SZPA. Characteristic absorption peaks were observed at 3420 cm^−1^ (O-H stretching vibration), 2920 cm^−1^ (C-H stretching of methylene groups), 1610 cm^−1^ (skeletal vibration of benzene rings), and 1480 cm^−1^ (aromatic ring stretching), confirming the methylene cross-linking reaction between the phenolic resin and HMTA. Additionally, peaks indicative of silicon-containing functional groups in the hybrid aerogel were identified: the Si-O-C stretching vibration at 980 cm^−1^, and the stretching and bending vibrations of Si-O-Si at 1110 cm^−1^ and 460 cm^−1^, respectively. Furthermore, the characteristic peaks at 867 cm^–1^ and 1370 cm^−1^ are assigned to the B-O stretching vibrations in B-O-Si and B-O-C structures, respectively [28].

The ^29^Si NMR spectrum of SZPA-4 was presented in Figure 2d. The chemical shifts observed at −95 and −103 ppm corresponded to Q2 [Si(OX)_2_] and Q3 [Si(OX)] silicon environments, where X represented H or an alkyl group. Specifically, the presence of Q2 and Q3 signals indicated that the SiO_2_ nanoparticles possessed surface groups, primarily consisting of alkoxy or hydroxyl functionalities. These groups might have undergone dehydration condensation reactions with hydroxyl groups from MTMS, KH-560, and boric acid, or formed hydrogen bonds with hydroxyl groups on the PR molecular chains. The chemical shift observed at −115 ppm is attributed to the Q4 structure of SiO_2_ nanoparticles, where silicon atoms were tetrahedrally coordinated with oxygen [24,29].

The chemical structure of SZPA-4 had been further characterized by XPS, as shown in Figure 2c. The survey scan revealed characteristic peaks at 534.1 eV for O 1s, 286.1 eV for C 1s, 194.0 eV for B 1s, 186.0 eV for Zr 3d, and 105.4 eV for Si 2p [14,23,30]. The high-resolution C 1s spectrum (Figure 2e) displayed five chemical states: π–π satellite, C–O–C, C–O–Si, C–C, and C–Si. The Si 2p spectrum (Figure 2f) was deconvoluted into four components corresponding to Si–C, Si–O–Si, and Si–O–B environments. When the silicon-containing component coexists with the zirconium-based particles in the physically dispersed composite system, the electron cloud density around the silicon atom will be weakly affected by the adjacent high electronegative zirconium particles, which will cause a slight peak splitting phenomenon near Si2p (103.5 eV), resulting in the fitting of the spectrum [30]. It is difficult to achieve a complete match between the fitting curve and the spectral characteristics. The O 1s spectrum (Figure 2g) exhibited three chemical states, which were assigned to Si–O–C, Si–O–Si, and –OH species. The B 1s spectrum (Figure 2h) was fitted to three peaks: B–O–C, B–OH, and B–O–Si. In the Zr 3d spectrum (Figure 2i), the binding energies at 182.72 eV and 185.10 eV were attributed to Zr–O 3d_3/2_ and Zr–O 3d_5/2_, respectively. The Zr 3d signal exhibited a weak peak intensity and a broadened peak shape. This phenomenon is primarily attributed to the incorporation of zirconia into the aerogel matrix via the physical dispersion approach employed in this work. In contrast to the chemical co-precursor method, which enables the homogeneous dispersion of zirconium species at the molecular scale, the physical blending of zirconia nanoparticles as fillers inevitably results in poor dispersion uniformity within the silica matrix, accompanied by localized particle agglomeration.

**Figure 3:**
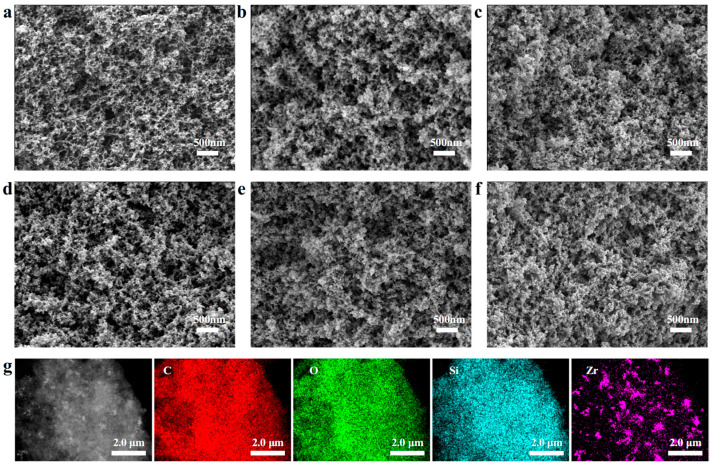
The morphological characteristics of PR and SZPA and the EDS spectrum of SZPA-4: (**a**) PR, (**b**) SZPA-1, (**c**) SZPA-2, (**d**) SZPA-3, (**e**) SZPA-4, (**f**) SZPA-5, (**g**) TEM images and EDS spectra of SZPA-4.

The micromorphology of the PR and SZPA was shown in Figure 3a–f. All samples exhibited a uniform and integral three-dimensional continuous network, composed of nanoscale gel particles and micron-sized clusters. The presence of macropores was essential for successful ambient-pressure drying, as they mitigated structural collapse induced by capillary forces during drying. Moreover, the hierarchical porous structure extended the heat transfer pathway, which can effectively improve the thermal insulation performance of SZPA. Figure 3g presents a Transmission Electron Microscopy (TEM) dark-field image and EDS element distribution of SZPA-4. The EDS results revealed that C, O, and Si were uniformly distributed, whereas Zr exhibited slight heterogeneity due to the agglomeration of zirconia nanoparticles. The structural homogeneity of the composite aerogel was attributed to the in situ formation of the hybrid network and strong intermolecular interactions between the SiO_2_ nanoparticles and phenolic resin molecular chains during synthesis.

**Figure 4:**
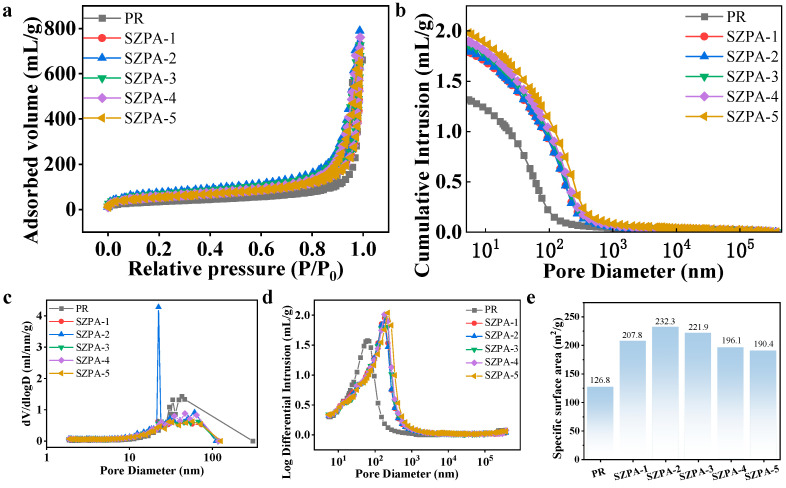
The pore structure characteristics of PR and SZPA: (**a**) nitrogen adsorption–desorption curve, (**b**) cumulative mercury intake, (**c**) BJH pore size distribution, (**d**) pore size distribution by mercury intrusion method, (**e**) BET specific surface area.

The pore structures of the PR and SZPA samples were further characterized by nitrogen adsorption–desorption analysis and mercury intrusion porosimetry. The adsorption–desorption isotherms, Barrett–Joyner–Halenda (BJH) pore size distribution curves, and Brunauer–Emmett–Teller (BET) specific surface area data are presented in Figure 4. According to the International Union of Pure and Applied Chemistry (IUPAC) classification, the aerogels exhibited Type IV isotherms (Figure 4a), with a distinct hysteresis loop in the relative pressure range of P/P_0_ = 0.8–1.0, indicating the presence of a mesoporous [31]. As shown in Figure 4c, the pore size distribution of PR is primarily concentrated in the range of 15–30 nm, while that of all SZPA mainly falls within 10–50 nm, confirming the mesoporous-dominated structure. Mercury intrusion porosimetry provided additional insights into pore characteristics. The intrusion curves reflected variations in pore size distribution, where a higher cumulative intrusion volume corresponded to higher porosity (Figure 4b). Incorporation of the inorganic phase significantly enhanced the porosity of the PR-based aerogels. The pore size distribution obtained from mercury intrusion (Figure 4d) revealed the formation of macropores in the SZPA, which were absent in the pure PR sample. The porosity values of PR and SZPA prepared by atmospheric pressure drying were 65.7%, 72.8%, 73.6%, 75.8%, 77.3%, 78.1%, respectively. PR samples only rely on the cross-linking of phenolic resin molecules to form an organic network. When dried at atmospheric pressure, the surface tension of the liquid phase can easily lead to network shrinkage and pore collapse, so the porosity is low. The SiO_2_-ZrO_2_ inorganic nanoparticles introduced into the SZPA will form an organic–inorganic interpenetrating network with the phenolic resin matrix. During the drying process, this composite skeleton can better resist the capillary force and reduce the collapse of the pores, thereby retaining more pores and increasing porosity. A skeleton morphology was observed by scanning electron microscopy. The PR skeleton was denser and the SZPA was more porous. Based on these analyses, it was concluded that the SZPA possessed a uniformly distributed hierarchical pore structure dominated by mesopores, which contributed to their enhanced thermal insulation performance.

### 2.3. The Mechanical Properties, Thermal Stability, and Thermal Insulation Properties

**Figure 5:**
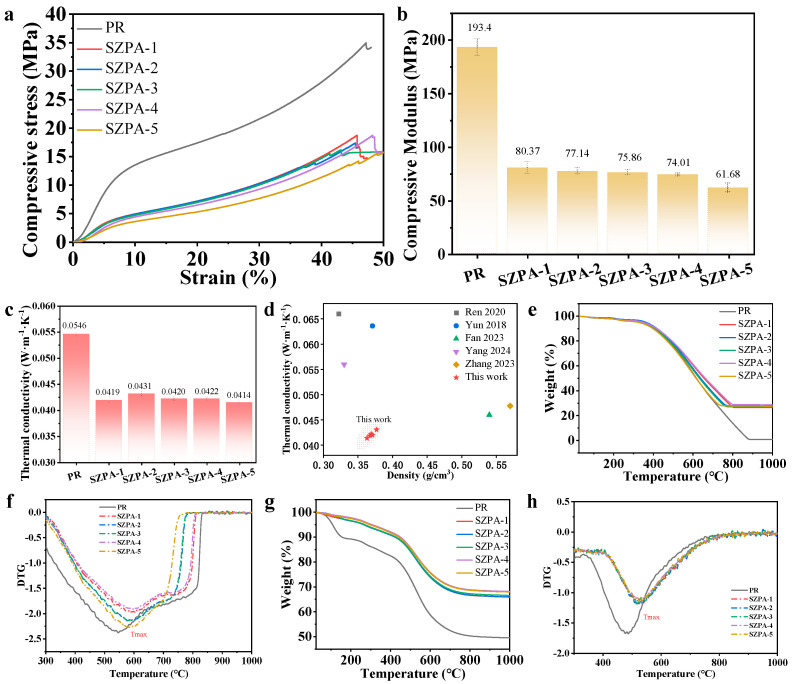
Mechanical and thermal insulation properties (**a**) Compressive stress–strain curves, (**b**) Compressive modulus, (**c**) Thermal conductivity, (**d**) Performance comparison between SZPA and organic–inorganic hybrid aerogels reported in the literature [21,28,32,33,34]. (**e**,**f**) Thermogravimetric analysis (TGA-DTG) in air atmosphere, (**g**,**h**) Thermogravimetric analysis (TGA-DTG) in argon atmosphere.

The mechanical properties of aerogels are critical for their practical applications. The compressive performance of PR and SZPA was evaluated, as shown in Figure 5a,b and Table 1. The stress–strain curves of all aerogels exhibited similar profiles, featuring three different deformation regions: linear elasticity, plastic yield, and densification. No samples showed brittle fracture in the strain range of 0–45%. The test results demonstrate that the compression modulus of PR is considerably higher than that of the SZPA-series composite aerogels. Silicon–zirconium composite modification significantly reduces the compressive strength of the material. This is because the introduction of nanoparticles alters the cross-linked network structure of phenolic resin, rendering the matrix more susceptible to deformation under compressive loading. With the increase in nano-zirconia content, the compression modulus of the material decreases progressively (from 80.37 MPa to 61.68 MPa). This finding indicates that as the zirconia content increases, the dispersibility of nano-zirconia within the composite system decreases; alternatively, the agglomerated zirconia particles disrupt the cross-linked network structure of the resin, ultimately resulting in the degradation of the material’s mechanical properties.

Thermal conductivity was a key performance parameter for aerogels in thermal protection applications, as it was closely related to their structural characteristics. As shown in Figure 5c, the thermal conductivities of PR and SZPA were 0.0546, 0.0419, 0.0431, 0.0420, 0.0422, and 0.0414 W/(m·K), respectively. Compared with PR, the thermal conductivity is reduced by about 24%. At the same time, As illustrated in Figure 5d, we compared the density and thermal conductivity of SZPA with those of other organic–inorganic hybrid aerogels fabricated via similar state-of-the-art processes. The results demonstrate that SZPA exhibits superior performance in these two key physicochemical properties [21,28,32,33,34]. According to the heat transfer theory, the effective thermal conductivity of aerogels consists of gas, solid, radiative, and convective contributions [35]. At low temperatures and under atmospheric pressure, radiative thermal conductivity became negligible when the pore size was smaller than 1 mm. Similarly, convective thermal conductivity could be disregarded at room temperature and pressure. Therefore, under ambient conditions, the thermal conductivity of aerogels was primarily governed by density and pore structure. The primary reason for the reduced thermal conductivity of the SZPA-series samples lies predominantly in the inherently low thermal conductivity of silicon oxide. As the primary framework component of the hybrid aerogel, silicon oxide can effectively suppress solid-phase heat transfer, thereby lowering the overall thermal conductivity of the material. The non-linear variation trend of thermal conductivity with composition (e.g., the difference in thermal conductivity between SZPA-2 and SZPA-4) arises from the incorporation of zirconia into the framework in the form of a filler. The addition of zirconia particles disrupts the continuity and heat transfer pathways of the silica-phenolic matrix, while the dispersion state of zirconia in different formulations (e.g., local agglomeration in certain formulations) also exerts a non-linear influence on the heat transfer process. The combined effect of these two factors accounts for the non-monotonic variation in thermal conductivity with increasing zirconia content.

As the silica and zirconia content increased, variations in shrinkage and solid content led to a fluctuating density trend: an initial decrease, followed by an increase, and then another decrease. Correspondingly, the pore volume and average pore size of the aerogels first increased and then decreased, reflecting concomitant changes in porosity. According to empirical models, effective gas-phase thermal conductivity was mainly influenced by porosity and average pore size [36]. Larger porosity and pore sizes resulted in higher effective gas thermal conductivity. The combined effect of gas- and solid-phase contributions ultimately caused the overall thermal conductivity of the aerogels to increase initially and then decrease.

The thermal stability and thermal decomposition behavior of PR and SZPA were systematically evaluated by thermogravimetric analyzer (TGA) combined with differential thermogravimetric (DTG) curve. The results are shown in Figure 5g,h (argon atmosphere) and Figure 5e,f (air atmosphere) [23,37]. In argon atmosphere, when the thermal decomposition temperature exceeds 200 °C, the mass residue rate of SZPA is significantly higher than that of PR: the mass residue of PR at 800 °C is 50.28%, and with the increase in ZrO_2_ addition, the mass residue of SZPA increases to 66.61%, 66.69%, 67.25%, 68.61% and 68.84%, respectively. Further analysis of the DTG curves showed that the thermal decomposition rate and the maximum thermal decomposition temperature (Tmax) of PR were much lower than those of the SZPA series. In the air atmosphere, the maximum mass residue of all samples was 28.73% (a sample of SZPA series), but the thermal decomposition rate of SZPA was significantly lower than that of PR, and the maximum thermal decomposition temperature (Tmax) was significantly improved. The results further confirmed the positive effect of inorganic phase modification: the inorganic–organic nanonetwork structure formed during the modification process, in which the cross-linked structure of Si-O-C and B-O-Si reduced the number of phenolic hydroxyl functional groups with poor thermal stability in the network, thus effectively improving the thermal stability and oxidation resistance of aerogels. Among the series, SZPA-4 exhibited the most balanced overall performance, combining high mass residue, superior thermal stability, low thermal conductivity, and adequate mechanical properties, making it the most suitable candidate for further investigation.

**Figure 6:**
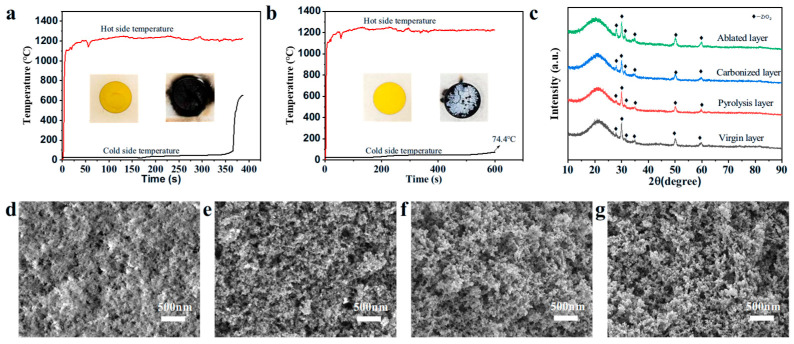
(**a**,**b**) The temperature distribution of hot and cold surfaces of PR and SZPA-4 after ablation for 10 min, (**c**) XRD patterns of different regions of SZPA, (**d**–**g**) the micro-morphology of SZPA-4 after ablation.

To evaluate the thermal insulation performance of SZPA in a high-temperature aerobic environment, PR and SZPA-4 samples (Φ50 mm × 20 mm) were subjected to a 1200 °C butane flame for 600 s, while the hot and cold surface temperatures were monitored using thermocouples. As shown in Figure 6a,b, both PR and SZPA-4 were exposed to the flame. After 300 s, the PR sample fractured due to excessive shrinkage, which was accompanied by a rapid rise in cold surface temperature. In contrast, the cold surface temperature of SZPA-4 rose gradually, reaching only 74.4 °C after 600 s, and the mass ablation rate of the sample is 2.67 × 10^−3^ g/s, and the linear ablation rate is 1.48 × 10^−3^ mm/s. The ablated surface of SZPA-4 appeared white, and the cross-section was divided into four distinct zones: the ablated surface, carbonized layer, pyrolysis layer, and virgin layer. In this study, the flame temperature of the butane spray gun was strictly controlled at 1200 °C, and the ablation test was carried out in an air atmosphere. Under this exper-imental condition, the thermodynamic conditions required for the formation of carbides (such as SiC) and zirconium silicate (ZrSiO_4_) are not satisfied. It can be seen from the X-ray Diffraction (XRD) diffraction patterns of different ablation regions in Figure 6c that nano-zirconia was successfully introduced into the SZPA-4 aerogel [38]. The ceramic layer formed on the ablation surface of the sample is mainly composed of the thermally stable crystalline phase of zirconia and the silica derived from the hybrid matrix. Figure 6d–g showed the microstructure of the ablated samples. The aerogel retained a porous skeleton structure, which effectively impeded heat transfer during ablation. After resin oxidation, a dense layer formed on the surface, which effectively blocked oxygen ingress. The microstructure of the pyrolysis zone closely resembled that of the virgin zone, indicating that SZPA-4 preserved its original pore structure and morphological characteristics under high-temperature conditions, thereby exhibiting efficient thermal insulation performance.

**Figure 7:**
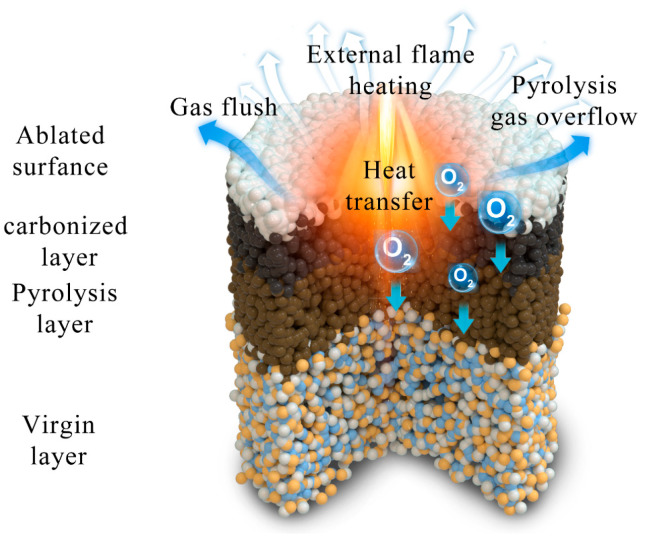
The schematic diagram of ablation and thermal insulation mechanism of SZPA.

Based on the aforementioned analysis, the ablation and thermal insulation mechanism of the SZPA are elucidated as follows (Figure 7). An extreme environment characterized by high temperature, aerodynamic erosion, and oxidation is created at the aerogel surface by the butane flame. The oxidation resistance of the matrix is significantly enhanced by the synergistic effect between silica and zirconia, thereby resulting in the preservation of a greater amount of pyrolytic carbon. On the ablated surface, a dense, multiphase ceramic layer with an island-like structure is formed, which acts as an effective thermal barrier, while aerodynamic erosion is resisted by this layer. Consequently, the ablation resistance of the hybrid aerogel is considerably improved by this structural evolution. The high-temperature ablation resistance and thermal insulation mechanism of SZPA-4 are summarized as follows [29,39]: (1) Upon exposure to a high-temperature flame, the ablated surface of SZPA-4 absorbed heat through ablation and decomposition, generating pyrolysis gases. The volatilization of these gases dissipated considerable heat, while the heat-blocking effect and the structural integrity of the aerogel within the boundary layer hinder further penetration of high-temperature oxidative airflow into the material. (2) Owing to the thermal barrier effect of the ablated surface, the carbonized layer of the aerogel was exposed to only limited oxygen, allowing the organic skeleton to undergo carbonization rather than complete oxidation. The retained porous structure further impeded heat transfer toward the inner regions of the sample. (3) The combined effects of the thermal barrier and carbonized zone prevent most of the ablation-induced heat and oxygen from propagating inward, resulting in the formation of a pyrolysis layer. The microstructure of this zone remained similar to that of the virgin layer, with no significant shrinkage or collapse, thereby providing additional resistance to heat transfer. (4) Throughout the ablation process, the virgin layer maintained a low-temperature, preserving the original morphology and structure of the aerogel. Thus, even under extreme conditions, SZPA-4 exhibited exceptional thermal insulation performance.

## 3. Conclusions

In summary, SiO_2_-ZrO_2_-phenolic resin aerogels were successfully fabricated via a stepwise hybridization strategy. The resulting aerogels exhibited low density, low thermal conductivity, high thermal stability, and excellent resistance to high-temperature oxidation. It achieved an ultralow thermal conductivity of 0.0414 W/(m·K) at room temperature, while the residual mass in an argon atmosphere was as high as 68.84%. When subjected to a 1200 °C butane flame for 600 s, SZPA-4 demonstrated outstanding ablation resistance and thermal insulation performance, with a cold-side temperature of only 74.4 °C. These findings confirmed that the stepwise hybridization strategy effectively improved the structural integrity and multifunctional performance of phenolic resin-based aerogels. This work therefore provided a feasible strategy for developing high-performance phenolic resin aerogels for thermal protection, showcasing significant application potential in both civilian and military fields.

## 4. Materials and Methods

### 4.1. Reagents and Materials

Silica sol (Solvent: ethanol system), solid content (20 wt.%), particle size (15 nm), manufacturer: Suzhou Xilika Electronic Materials Co., Ltd. (Suzhou, China). Methyl trimethoxysilane (MTMS), purity: analytical grade, manufacturer: Shanghai McLean Biochemical Technology Co., Ltd. (Shanghai, China). γ-(2,3-epoxypropoxy)propytrimethosysilane (KH-560), manufacturer: Jiangxi Chenguang New Materials Co., Ltd. (Jiujiang, China). Hexamethylenetetramine (HMTA), manufacturer: Chengdu Kelong Chemical Co., Ltd. (Chengdu, China). Nano-zirconia dispersion (solvent: ethanol system), solid content (20 wt.%), particle size (30 nm), manufacturer: Hangzhou Jiupeng New Materials Co., Ltd. (Hangzhou, China). Ethanol (EtOH), purity: analytical grade, manufacturer: Tianjin Fuyu Fine Chemical Co., Ltd. (Tianjin, China). Boric acid, purity: analytical grade, manufacturer: Sinopharm Chemical Reagent Co., Ltd. (Shanghai, China). Phenolic resin (PR), manufacturer: Kunyi Resin Materials Technology Co., Ltd. (Jiangmen, China).

### 4.2. Preparation of SiO_2_-ZrO_2_-Phenolic Aerogel (SZPA)

Detailed information on the sample compositions is presented in Table 2. With the mass ratio of PR:EtOH = 1:2, PR was dissolved in EtOH solution by mechanical stirring to obtain PR sol. A high-purity silica sol was mixed with a zirconia dispersion to form a silica–zirconia sol. The formulations used a fixed silica sol-to-PR mass ratio of 0.4:1 and varying zirconia-to-PR mass ratios (0.01:1, 0.03:1, 0.05:1, 0.07:1, 0.1:1). The MTMS was weighed according to a certain proportion, poured into the silicon–zirconium sol, and stirred at room temperature for 12 h. Then, KH-560 and sol were slowly dropped into the mixed sol at a mass ratio of 0.01:1, and magnetically stirred for 2 h. Then, boric acid was added to the mixed sol, the mass ratio was 0.01:1, and the magnetic stirring was 2 h. The silicon–zirconium sol with different zirconia content was added to the PR sol and sheared at high speed for 10 min. The dosage of HMTA was 20% of the mass of PR. The resulting mixture was poured into a mold (Φ55 mm × 50 mm cylindrical mold), and then the mold was transferred to a sealed metal container and cured at 90 °C for 48 h. After curing, aerogel samples with complete porous structures were obtained based on solvent volatility. We took out the wet gel and dried it in an oven at 60 °C and 80 °C for 24 h to obtain SZPA. The resulting samples were labeled SZPA-1 through SZPA-5. For comparison, pure phenolic resin aerogels without silica or zirconia were also prepared following a similar procedure, omitting the addition of the silica–zirconia sol.

### 4.3. Sample Characterization

The microstructure and morphology were characterized by Scanning Electron Microscopy (SEM, Zeiss Sigma 300, Zeiss, Oberkochen, Germany) and Transmission Electron Microscopy (TEM, JEOL JEM-F200, JEOL, Tokyo, Japan). The nitrogen adsorption–desorption curve of the sample was obtained by the gas adsorption method, Brunauer–Emmett–Teller (BET, Best 3H-2000PM2, Best, Beijing, China), and the pore size distribution of the aerogel was tested by Autopore V 9605 mercury porosimeter (Micromeritics, Norcross, GA, USA). The density was determined by measuring the mass and size of the aerogel. The characteristic groups of aerogels were analyzed by a Vertex 70 infrared spectrometer (FTIR, Bruker, Billerica, MA, USA). The chemical environment of 29Si in the aerogel was analyzed by an Agilent 600M nuclear magnetic resonance spectrometer (NMR, Agilent, Santa Clara, CA, USA). X-ray Photoelectron Spectroscopy (XPS, Thermo Fisher Scientific, Waltham, MA, USA) was used to test the chemical bonding of aerogels. The samples were heated from room temperature to 1000 °C at a heating rate of 5 °C min^−1^ in argon and air atmospheres, respectively, using a thermogravimetric analyzer (STA 449F3, NETZSCH, Selb, Germany). The thermal decomposition characteristics and mass residual rate of aerogels were analyzed. The crystal form of aerogel was analyzed by Bruker D8 Advance X-ray diffractometer (XRD, Bruker, Bremen, Germany). The FL 4204 GL universal testing machine (Fule Test Technology, Shanghai, China) was used to evaluate the compressive strength at a speed of 1 mm·min^−1^, the samples were prepared with dimensions of 20 × 20 × 20 mm, and the tests were performed in triplicate. The thermal conductivity of PR and SZPA at room temperature was measured using a Hot Disk thermal constant analyzer (TPS 2500 s, Hot Disk, Gothenburg, Sweden). The high-temperature thermal insulation performance of SZPA-4 was evaluated using a 1200 °C butane flame test, with surface and back temperatures monitored via thermocouples (R type platinum rhodium corundum thermocouple, Yancheng Dikwei Measurement and Control System Co., Ltd., Yancheng, China). Sample dimensions (50 mm in diameter × 20 mm in thickness), distance between the spray gun nozzle and the sample surface (10 cm).

## Figures and Tables

**Table 1:** Compressive strength of PR and SZPA.

Sample	Compressive Strength (MPa)		
	3% ε	5% ε	10% ε
PR	4.49	8.57	13.54
SZPA-1	1.79	3.26	4.95
SZPA-2	1.70	3.07	4.92
SZPA-3	1.62	3.02	4.74
SZPA-4	1.05	2.52	4.42
SZPA-5	0.98	2.10	3.62

**Table 2:** PR and SZPA component information.

Samples	Silica Sol (mL)	Nano-Zirconia (mL)	MTMS (mL)	KH-560 (mL)	Boric Acid (g)	PR (g)	EtOH (mL)	HMTA (g)
PR	/	/	/	/	/	48	120	9.6
SZPA-1	100	2.5	5.6	0.5	1.5	48	120	9.6
SZPA-2	100	7.6	5.6	0.7	1.8	48	120	9.6
SZPA-3	100	12.6	5.6	0.9	2.0	48	120	9.6
SZPA-4	100	17.7	5.6	1.2	2.5	48	120	9.6
SZPA-5	100	25.3	5.6	1.5	2.5	48	120	9.6

## Data Availability

The data presented in this study are available on request from the corresponding authors.
